# Application of Metagenomic Next-Generation Sequencing in the Diagnosis of Infectious Keratitis

**DOI:** 10.1155/2024/9911979

**Published:** 2024-04-29

**Authors:** Xin-Yu Pan, Meng Wang, Yi-Dan Xu, Lin-Nong Wang

**Affiliations:** Department of Ophthalmology, Nanjing First Hospital, Nanjing Medical University, Nanjing, Jiangsu Province 210006, China

## Abstract

**Purpose:**

To determine the advantages of next-generation metagenomic sequencing (mNGS) technology in the diagnosis and treatment of infectious keratitis (IK).

**Methods:**

A total of 287 patients with IK admitted to the Department of Ophthalmology of Nanjing First Hospital between August 2018 and December 2022 were analyzed retrospectively, and the pathogenic causes, etiological characteristics, detection, treatment methods, and efficacy were summarized.

**Results:**

Trauma and foreign matter were the most common causes of IK (144 patients, 50.2%). Of the 287 patients, 228 (79.4%) were diagnosed with a specific etiology, including 110 (48.2%) fungal infections, 44 (19.3%) viral infections, 42 (18.4%) mixed infections, and 30 (13.2%) bacterial infections. Filamentous fungi represented by *Fusarium* and *Aspergillus* were the most common, followed by bacteria such as *Pseudomonas aeruginosa*, *Streptococcus pneumoniae*, viruses (Herpes Simplex Virus/Varicella-Zoster Virus), and parasites. The positivity rates of secretion culture, corneal laser confocal microscopy (CM), mNGS, and pathological sections were 47.3% (133/281), 45.3% (111/245), 83.9% (104/124), and 19.3% (40/207), respectively. The positivity rate of mNGS for bacteria and viruses was higher than that of the other methods, and the positivity rate for fungi was the same as that for CM. As a result, 214 cases (74.6%) were cured, 51 cases (17.8%) improved, 8 cases (2.8%) did not heal, ocular content enucleation was performed in 14 cases (4.9%), and the overall efficacy rate was 92.3%.

**Conclusion:**

Trauma and foreign matter are the main causes of IK. The mNGS technology is an efficient and comprehensive detection method for viruses and bacteria, especially for mixed infections.

## 1. Introduction

Infectious keratitis (IK) stands as a prominent cause of blindness in China and is ranked first among the causes of corneal blindness [1, 2]. Its global incidence ranges from 2.5 to 799 cases per 100,000 individuals per year. Risk factors such as contact lens wear, trauma, ocular surface and eyelid diseases, and eye surgery have been shown to be major causes of IK [3]. Bacteria, fungi, viruses, and parasites are pathogenic microorganisms, and mixed infections can rapidly develop into corneal ulcers, perforations, endophthalmitis, and scars that are detrimental to vision [4, 5]. Although various bacterial species have been associated with bacterial keratitis, gram-positive *Staphylococcus aureus* and gram-negative *Pseudomonas aeruginosa* are the most common pathogens [6]. Specifically, the prevalence of *S. aureus* keratitis is 8–36% in all cases of bacterial keratitis, whereas the prevalence of *P. aeruginosa* as the causative organism of keratitis is approximately 10–39% [6]. Additionally, fungi (such as *Aspergillus* spp., *Candida* spp., and *Fusarium* spp.), viruses (such as herpes simplex virus [HSV]), and parasites (such as *Acanthamoeba*) are responsible for infectious keratitis [7]. Therefore, early identification of the pathogen, treatment with effective drugs, and surgical intervention are important for the successful management of patients with IK.

The sensitivity of traditional microbial diagnostic techniques such as secretion smears and cultures for pathogens is limited. Confocal microscopy is sensitive for mycelia and *Acanthamoeba* but identifying fungal populations using it is difficult [8]. Metagenomic next-generation sequencing (mNGS) has been rapidly and extensively applied for pathogen detection [9, 10]. Compared with traditional pathogen detection methods, mNGS can quickly detect various of pathogens and identify a large variety of pathogens [11, 12]. Borroni et al. [13] used shotgun metagenomic analysis to assess the microbiota of culture-negativeCorneal Impression Membrane microbial keratitis samples and found that shotgun sequencing could be used as a diagnostic tool for microbial keratitis samples. Another study reported that *Capnocytophaga*, a rare and aggressive infection, could not be identified using traditional culture methods but could be detected using metagenomic deep sequencing [14]. Greenwald et al. [15] demonstrated the diagnostic potential of metagenomic deep sequencing for identifying post-LASIK keratitis. A recent study used mNGS to demonstrate *Corynespora cassiicola* as the cause of fungal infection, thus establishing the groundwork for further treatment of the patient [16]. These reports suggest that mNGS may not only be a novel diagnostic tool for the diagnosis of refractory IK but can also facilitate the rapid identification and timely management of IK that is difficult to diagnose.

In this study, mNGS was used to diagnose IK. The causes and characteristics of pathogenic microorganisms in 287 patients (288 eyes) were retrospectively studied. mNGS for the clinical diagnosis of IK was evaluated and compared with various traditional detection methods. This study provides a reference for the clinical diagnosis and treatment of IK.

## 2. Methods

### 2.1. General Information

Between August 2018 and December 2022, 287 consecutive cases (288 eyes) of IK that were admitted and treated at the Department of Ophthalmology of Nanjing First Hospital (Nanjing, China) were enrolled, including 184 males (184 eyes) and 103 females (104 eyes). The average age was 62 years (range, 53–70 years), and the average duration of hospitalization was 12 days (range, 8–16 days). The pathogenic factors, etiology, detection methods, treatment, and outcomes were analyzed. The inclusion criteria were diagnosed as IK [17], and the exclusion criteria were shown as follows: (1) keratitis caused by noninfectious factors, such as sericultural keratitis, allergic keratoconjunctivitis, superficial punctate keratitis, filamentous keratitis, sclerosing keratitis, graft-versus-host disease, as well as neurotrophic keratitis, exposure keratitis, drug-induced keratitis, and corneal cicatricial ulcer; (2) patients with uncontrolled mental illness and consciousness disorder; (3) pregnant females. This study was reviewed and approved by the Ethics Committee of the Nanjing First Hospital, Nanjing Medical University (approval no. KY20230424-04-KS-01), and all the patients provided informed consent. All the experiments were performed in accordance with the principles of the Declaration of Helsinki.

### 2.2. Diagnosis

A preliminary diagnosis was made based on the patient's history, symptoms, signs of IK, and laboratory examination. After admission, a series of laboratory tests were performed, and the diagnosis was revised. These tests included (1) secretion culture (SC), identification, and drug sensitivity test: secretion of conjunctival sac, ulcer margin tissue, and aqueous humor were collected at the first visit, after admission, and during surgery; (2) corneal scraping microscopy (CS) performed using a potassium hydroxide wet tablet method [18]; (3) corneal laser confocal microscopy (CM) [19]: repeat testing was performed when fungal or parasitic infection was highly suspected and all other tests were negative; (4) pathological section (PS) [20]: lesion cornea was examined using routine hematoxylin-eosin (HE) and periodic acid-schiff (PAS) staining using OCUFACE biological microscopy. (5) mNGS [21, 22]: samples were collected during lesion debridement or keratoplasty, including the aqueous humor, ulcerated marginal tissue, and cornea (avoid specimen contamination). Among the SC, identification, and drug sensitivity tests, CS, CM, and PS were routine tests, and mNGS was also a routine test unless the patient refused.

### 2.3. mNGS Analysis

The collected samples were subjected to DNA extraction and purification using the QIAamp DNA Micro Kit (QIAGEN, Hilden, Germany). The concentration and quality of the isolated DNA were determined using a Qubit 4.0 (Thermo Fisher Scientific, MA, USA) and Qsep-1 (Bioptic Inc., Taiwan, China), respectively. Then, the DNA libraries were constructed using QIAseq™ Ultralow Input Library Kit (QIAGEN, Hilden, Germany), including fragmentation, end-repair, adapter ligation, size selection, and PCR enrichment and cleanup. Finally, the samples were sequenced on the NextSeq 550 platform (75 bp single-end reads) (Illumina, San Diego, USA). Raw sequencing data were subjected to qualified controls, and clean data were obtained. Then, human reads were removed by mapping reads to the human reference genome using SNAP software, and the remaining reads were aligned to the Microbial Genome Databases (https://ftp.ncbi.nlm.nih.gov/genomes/) using Burrows-Wheeler. In each batch of samples, both negative and positive controls were set up, and the same procedures were used for mNGS detection and bioinformatic analysis [16]. The number of specific reads per million (RPM) for each detected pathogen was calculated. A positive mNGS result was defined when the microorganism was not detected in the negative control (“no template” control), and the genome coverage of the detection sequence ranked top 10 among the same type of microorganisms, or when the ratio of RPMsample to RPMNTC was (RPMsample/RPMNTC) > 10 if the RPMNTC≠ 0.

Interpretation of the report: (A) Detection of the pathogenic HSV/Varicella-Zoster Virus (VZV) sequences: Because current studies have not found HSV/VZV colonization on healthy human eye surfaces [23, 24], it was judged as positive regardless of the number of sequences for HSV/VZV. The same was true for the detection of parasites. (B) Detection of pathogenic fungi or bacteria: if consistent with other detection methods, it was judged as positive; otherwise, combined with the medical history and clinical manifestations and the corresponding antifungal/bacterial treatment being effective, if consistent, it was judged as positive (C). Owing to the relatively low detection rate for intracellular and *Firmicutes* such as *Mycobacterium tuberculosis* and *Legionella*, and fungi [25], a low sequence number cannot be ruled out as a non-pathogenic pathogen. (D) No detection of pathogenic microorganisms, but background bacteria with higher sequences: referring to previous studies on background bacteria [23, 26, 27], the abnormal pathogen sequences detected were judged as positive if they were consistent with clinical practice and effective in the corresponding treatment. In our mNGS report, common background bacteria included *Propionibacterium acnes*, *Acinetobacter lwoffii*, *Acinetobacter johnsonii*, and *Staphylococcus epidermidis*, all of which belong to the common conjunctival sac bacteria group. Fungal keratitis (FK) can be accompanied by changes in the ocular surface bacterial community structures, and many background bacteria are highly abundant; therefore, attention should be paid [28].

Based on the above series of examinations, an etiological diagnosis was established, and the criteria were as follows (one of them met):

1. SC positivity: currently, it is the gold standard method of etiological diagnosis;
2. Fungal mycelium detected using CS/PS: scrape staining is also one of the gold standard method for fungal diagnosis [29].
3. CM shows typical fungal mycelia, *Acanthamoeba* cysts, or trophozoites [30], and the history and clinical manifestations of the cases are consistent with fungal/*Acanthamoeba* infections [31]. Antifungal/*Acanthamoeba* treatments were effective.
4. Consistent with the history and clinical manifestations of viral infection [31] and antiviral treatment was effective, other microbial tests were negative;
5. Positive mNGS.

### 2.4. Treatment

After the secretion culture method detected bacteria, drug sensitivity tests were performed, among which the positive results were shown as follows: (1) 27 cases of bacteria: *S. aureus*, *Streptococcus sanguineus*, *S. epidermidis*, *Streptococcus pneumoniae*, and *Chryseobacterium indologenes* were more sensitive to levofloxacin; *P. aeruginosa*, and *Acinetobacter junii* were more sensitive to tobramycin; and *Nocardia*, *Escherichia coli*, *Klebsiella pneumoniae*, and *Serratia marcescens*, *Enterobacter cloacae* were more sensitive to amikacin and cotrimoxazole; and *Morgenella Morganii* was more sensitive to amikacin; and *Cupriavidus* were sensitive to amikacin, tobramycin, and levofloxacin, whereas *Stenotropicomonas maltophilia* and *Delfteria acidalis* were sensitive to levofloxacin and cotrimoxazole. (2) 20 cases of fungi:17 cases of *Aspergillus* were resistant to fluconazole, and 3 cases of *Candida albicans* were sensitive to voriconazole, itraconazole, fluconazole, and amphotericin B. If sensitive to broad-spectrum antimicrobials, the original treatment regimen was maintained; otherwise, the sensitive antimicrobials were replaced, generally choosing two topical drugs +1 systemic drugs, and then using atropine mydriasis and tacrolimus anti-inflammatory as appropriate.

Based on preliminary diagnosis, patients with undefined pathogenic microorganisms were administered broad-spectrum antimicrobial therapies. After identification of the microorganisms, targeted treatment was administered, and drug-sensitive populations were treated with sensitive antibiotics. The drug routes and treatment methods were as follows: (1) bacterial infection was treated with fluoroquinolones such as moxifloxacin, levofloxacin, or gatifloxac eye drops; gatifloxacin gel, tobramycin, or ofloxacin eye cream; and cephalosporin three-generation intravenous infusion. Viral infection was treated with topical ganciclovir gel, oral acyclovir, or ganciclovir capsules, and hormones (flumirone eye solution) was combined for stromal keratitis and endotheliitis. Fungal infection was treated with natamycin, voriconazole eye drops, oral itraconazole, or voriconazole capsules and chlorhexidine eye liquid was used for *Acanthamoeba* infection. (2) Corneal debridement and stromal anterior chamber drug injection (S-ACDI) were treated with 0.1 mL of 1% vancomycin for gram-positive bacteria, 2.25% ceftazidime for gram-negative bacteria, and 0.25% voriconazole for fungi. (3) Keratoplasty (KP) for those whose inflammation was not controlled even developed into deeper after 1-2 weeks of treatment. Deep lamellar keratoplasty was performed in patients without full-layer corneal invasion, and penetrating keratoplasty was performed in patients with full-layer corneal invasion. (4) Enucleation of eye contents (ECE) for those whose inflammation spread into the eye and could not be controlled or was accompanied by the loss of eye contents; and (5) others such as conjunctival flap covering, vitrectomy.

Slit-lamp examination and corneal fluorescein staining were performed every 2 weeks after discharge for 3 months. The efficacy evaluation criteria were as follows: (1) cured: corneal ulcer healed, cornea, or graft were structurally intact, fluorescein staining was negative, anterior chamber reaction and abscess disappeared; (2) improvement: the area of corneal ulcer reduction >50%, and abscess of the anterior chamber was significantly reduced; (3) eye contents were enucleated; and (4) no cure: ulcer area reduction <50%, fluorescein staining was positive, and no change in the abscess of the anterior chamber.

### 2.5. Statistical Analysis

SPSS 27 software was used for statistical analysis. Data that did not conform to a normal distribution are represented as medians (interquartile range, IQR), and non-parametric tests were adopted. The statistical data were expressed as rate (*n*/%), using the *χ*^2^ or Fisher's exact test. The test level was *α* = 0.05.

## 3. Results

### 3.1. Diversity in Pathogenic Factors

**Figure 1:**
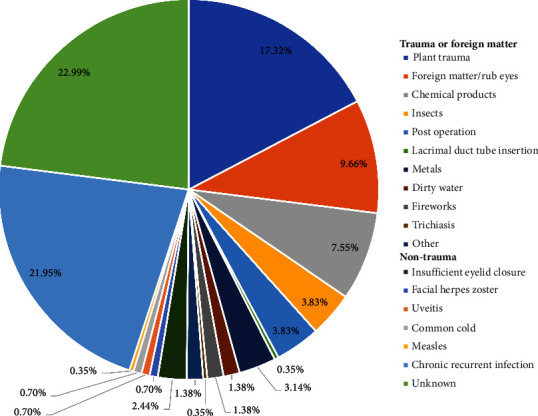
Pathogenic causes of 287 infectious keratitis, including trauma and non-trauma causes.

The predisposing factors for the 287 IK cases were diverse and included trauma and non-trauma causes. Among these, 144 (50.17%) were induced by trauma, including plant trauma (*n* = 50, 17.32%), foreign matter/rub eyes (*n* = 28, 9.66%), chemical products (*n* = 22, 7.55%), insects (*n* = 11, 3.83%), metals (*n* = 9, 3.14%), and firework (*n* = 4, 1.38%) (Figure 1 and Table S1). In addition, 63 of the 143 patients with non-traumatic IK (49.83%) had long-term chronic recurrent keratitis (21.95%), and some of them were sensitive to antiviral therapy (Figure 1 and Table S1).

### 3.2. Diverse Pathogenic Microorganisms

**Figure 2:**
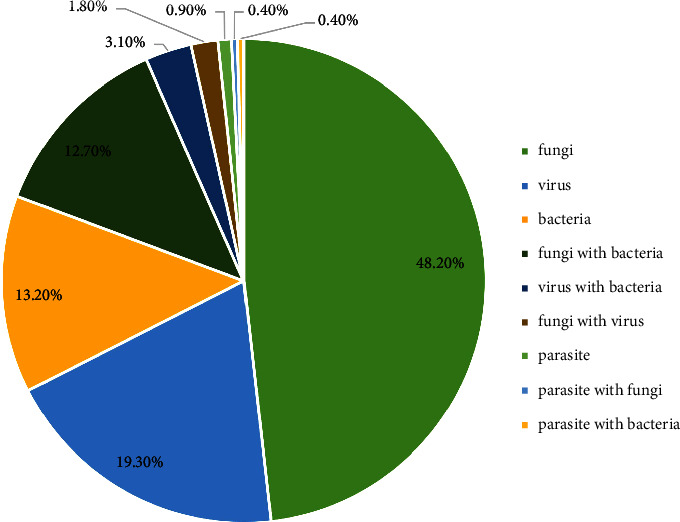
Pathogenic microorganisms in 228 cases of infectious keratitis.

Among the 287 patients with IK, 228 (79.4%) were diagnosed by etiology, and the composition ratio of pathogenic microorganisms is shown in Figure 2. Nearly half of the infections were fungal (48.2%), followed by viral (19.3%), bacterial (13.2%), mixed (18.4%), and parasitic (0.9%). Mixed infections (42, 18.4%) were mainly fungi combined with bacteria, followed by viruses combined with bacteria, and fungi combined with viruses (Figure 2). Additionally, a total of 125 strains of fungi, 76 strains of bacteria, 32 strains of viruses, and 4 strains of parasites (including mixed infections) were detected in 228 cases of IK.

A total of 125 fungal strains were detected in 144 cases of FK (including mixed infections). Filamentous fungi (94.4%), including *Fusarium* (36.8%), *Aspergillus* (24.8%), *Alternaria* (4.8%), *Penicillium* (4.0%), and *Cladosporium* (4.0%), were the most common. Yeast-like fungi (1.6%) and yeasts (3.2%) were rare (Table S2). Thirty-one cases of unknown species were confirmed based on the mycelia found in SC/CM/PS.

Seventy-six bacterial strains were detected in 67 cases of bacterial keratitis (BK) (including mixed infection). The most common bacteria were *Pseudomonas* (19.7%), *S. pneumoniae* (18.4%), *S. epidermidis* (10.5%), and *S. aureus* (6.6%). Among which gram-negative bacteria accounted for slightly more than gram-positive bacteria (Table S3).

A total of 32 strains of the virus were detected in 55 cases of viral keratitis (VK) (including mixed infections), which mainly included HSV-1 (96.9%), followed by VZV (3.1%). Twenty-three cases of unknown viral strains were diagnosed based on clinical manifestations (Table S4).

Four parasitic strains were detected in four cases of parasitic keratitis (PK) (including mixed infections). Of which, three cases were of *Acanthamoeba*: one case detected using CM without mNGS, one detected using CM and mNGS, and one detected by CM but not detected by mNGS. The remaining one case was of *Nematode* (Table S4).

### 3.3. Optimal Positive Rate of mNGS

The positive detection rates of fungi using the four methods were 69.2% for SC, 82.5% for CM, 72.9% for mNGS, and 36.7% for PS with statistical differences (*p* < 0.001, Table 1).

The positive rates of bacteria using the two detection methods were as follows: SC, 68.7% and mNGS, 89.5%, with a statistically significant difference (*p* = 0.016). In addition, pathogenic bacteria were detected using mNGS in 21 SC-negative cases, which combined with the specific clinical manifestations of bacterial infection: rapid onset, rapid development, severe irritation symptoms, and excessive purulent secretion; therefore, the mNGS report was accepted (Table 1).

The 287 patients were divided into two groups based on whether they were evaluated using mNGS. The diagnostic rate of the mNGS group was higher (92.7%) than that of the non-mNGS group (69.3%), as was the diagnostic rate of bacteria (30.6% vs. 17.8%), viruses (27.4% vs. 12.9%), and mixed infections (24.2% vs. 7.4%) but not that of fungi (56.5% vs. 45.4%) (Table 2).

The combined positivity rates of the four methods were 47.3% for SC, 45.3% for CM, 83.9% for mNGS, and 19.3% for PS. The positivity rate for mNGS was the highest (*p* < 0.001), as shown in Table 3.

The pathogenic causes in 59 patients with negative microbiological study results were as follows: 23 traumatic injuries caused by foreign matter, 5 with a perennial recurrent history, 3 with a history of eye surgery, and 1 with a history of chronic uveitis. CS was not performed in 56 cases, mNGS in 51, PS in 23, and CM in 13. Seven patients were admitted in severe condition, of whom two were recommended to be transferred to the hospital, five were subjected to ECE, and the remaining 52 showed improvement with empirical antiinfective therapy.

### 3.4. Treatment Methods and Efficacy

Various surgical methods were used in 287 patients, including KP (203 patients, 70.7%) and ECE (14 patients, 4.9%) (Table 4). After 3 months of follow-up, 214 cases (74.6%) were cured, 51 cases (17.8%) improved, 8 cases (2.8%) were not cured, and ECE was performed in 14 cases (4.9%). The overall efficacy rate (cure + improvement) was 92.3%.

## 4. Discussion

IK has a high incidence rate, seriously endangers human visual health, is often misdiagnosed, and results in delayed treatment [32]. To preserve the eyeball and restore visual function, identifying the causes and pathogenic microorganisms of IK are necessary [33, 34]. Standardized etiological detection is the most basic manifestation of accurate diagnosis of infectious diseases.

Our study revealed that IK mainly occurs in middle-aged and older adult males, primarily due to trauma or foreign matter in the eye, among which plant trauma accounted for 34.7%, which may be related to agricultural activities [35]. It was found the fungi were mainly represented by filamentous fungi, such as *Fusarium*, *Aspergillus*, *Alternaria*, *Penicillium*, and *Cladosporium*; and the most common bacteria were *P. aeruginosa*, *S. pneumoniae*, *S. epidermidis*, and *S. aureus*; and the viruses were mainly HSV-1 and VZV as well as the main parasites were *Acanthamoeba*, which were consistent with previous reports [1, 36].

The etiological diagnosis was confirmed in 228 patients with IK, among which fungal infection (48.2%) accounted for the largest proportion, followed by viruses (19.3%) and bacteria (13.2%), which differed from a previous report that bacteria are the most common causative agent [36]. The incidence of FK has recently increasing [37]. Compared with other pathogens, FK has a longer disease course, is more difficult to treat, and has a poor prognosis [8]. In our study, there were 42 cases (18.4%) of mixed infections, mainly fungi combined with bacteria, which usually occur after plant trauma to the eye. The irritation symptoms were severe and the fungal manifestations were easily concealed by bacterial infections [38]. Therefore, timely and comprehensive detection of all pathogenic microorganisms is the key to successful treatment.

Among the etiological testing methods, SC with good specificity remains the gold standard method of diagnosis, but its positive rate is limited, ranging from 32.7 to 79.4% due to the adverse influences of sampling site, timing, method, and medication history [29]. In this study, the positivity rate of SC was 47.3%, based on multiple samplings and examinations of each patient. CM has significant performance in the identification of fungal mycelia and *Acanthamoeba*; however, it is difficult to identify bacteria and viruses because of its lack of specificity. CS/PS is also considered one of the gold standard method for diagnosis. Fungi manifest as clusters of spores and mycelia in the matrix, whereas other microorganisms have no specific features [39]. PS can only be detected after KP or ECE and cannot be diagnosed at an early stage. Therefore, it is generally used for retrospective analyses and has limited therapeutic value.

In this study, the positivity rate of mNGS was higher than that of the other detection methods, especially for the detection of viral, bacterial, and mixed infections. PCR is also commonly used for viral detection; however, its disadvantage is that it can only detect known pathogenic microorganisms, and clinical suspicion is required in advance to determine the primers to detect suspected microorganisms [40]. However, mNGS has the potential to improve diagnostic rates because it is essentially unbiased and hypothesis-free [41]. The current study confirmed a higher diagnostic rate in the mNGS group than in the non-mNGS group. In addition to the high positivity rate of mNGS in fungal infections, which is similar to that of CM, mNGS can also identify fungal species. In the absence of CM and PS, mNGS improves the detection rate of bacterial infections. mNGS may be a relatively efficient method for treating eye diseases caused by unknown pathogenic microorganisms, particularly mixed infections.

For parasites, mNGS can be used to detect *Acanthamoeba* infections. *Acanthamoeba*, an emerging pathogen, is known to cause keratitis and is often reported in contact lens wearers and individuals with eye trauma [42]. *Acanthamoeba* cysts are reportedly resilient to disinfectants, antigenic animal drugs, and nutrients, posing a significant challenge to patient care [43]. *Acanthamoeba* keratitis is difficult to diagnose, and effective treatment options are limited [44]. Rammohan et al. [45] showed that over a 5 year period, 85 cases of culture-positive *Acanthamoeba* keratitis were identified (43 of them being co-infections); *Fusarium* was the most commonly identified species, followed by *Aspergillus*, and *Pseudomonas* was the most common bacterial isolate, which was in accordance with our results. Recent studies have used sequencing, fluorescence in situ hybridization (FISH), and corneal scrape microbiology and found that most clinical isolates of *Acanthamoeba* contain intracellular microorganisms (such as *P. aeruginosa*), which may affect the clinical characteristics of *Acanthamoeba* keratitis [46, 47]. Taken together, CM combined with mNGS can be effectively employed to identify *Acanthamoeba* keratitis; however, this conclusion needs to be verified using a larger sample size.

The advantages of mNGS in the diagnosis of IK are as follows: (1) Small samples are applicable. One of the main limitations in the diagnosis of eye diseases is the difficulty in obtaining a large number of samples. The mNGS method can characterize all genome contents, and even if the available sample size is limited, sometimes even <2 ng/mL of extracted DNA [41] or 0.1 mL of aqueous humor can be tested. (2) Rapid: The fastest return of results on the day after sampling is conducive for early diagnosis. (3) Drug-resistance genes can be further analyzed and confirmed using corresponding antibiotic sensitivity tests to guide clinical drug use.

However, this study has some limitations. For mNGS, there is no technical standard for eye disease diagnosis [40], and mNGS reports need to be interpreted with clinical judgment. In addition, it is inefficient for pathogens with cell walls (such as thick-walled fungi, tuberculosis) and must be counteracted with reagents to destroy the cell walls [25]. In addition, our research was retrospective, and some conclusions need to be confirmed with a larger sample size. There were a few cases of corneal scrape microscopy and amoebic keratitis, and no statistical analyses were performed. Finally, it was difficult to establish a non-infectious control group; therefore, it was impossible to analyze the sensitivity, specificity, and positive/negative predictive values of mNGS.

## 5. Conclusion

In conclusion, trauma and foreign matter are the main causes of IK. Fungi were the main pathogens, followed by viruses and bacteria. mNGS, which is conducive to an accurate diagnosis combined with clinical characteristics and other detection methods, is an efficient and more comprehensive detection method for viral, fungal, bacterial, and parasitic infections, especially for mixed infections. This study may provide information for improving the combination of clinical acumen and laboratory tests for etiological diagnosis and customized treatment without losing time.

## Figures and Tables

**Table 1:** Comparison of detection methods for fungus and bacteria.

Microorganisms	Detection methods	Sample case (*n*)	Positive case (*n*)	Positive rate (%)
Fungi	SC	143	99	69.2
	CM	137	113	82.5
	mNGS	70	51	72.9
	PS	109	40	36.7
	*χ* ^2^	60.436		
	*p*	<0.001		

Bacteria	SC	67	46	68.7
	mNGS	38	34	89.5
	*χ* ^2^	5.792		
	*p*	0.016		

SC, secretion culture; CM: corneal laser confocal microscopy; PS: pathological section; mNGS, metagenomic next-generation sequencing. For fungus: the positive rate of CM was higher than that of SC (*χ*^2^ = 6.681, *p*=0.010), but no difference was observed between CM and mNGS (*χ*^2^ = 2.608, *p*=0.106), the positive rate of SC was similar to mNGS (*χ*^2^ = 0.297, *p*=0.586), and the positive rate of PS was the lowest (all *p* < 0.05). For bacteria, 21 SC cases were negative but were detected using mNGS. Four cases of mNGS were negative but were detected using SC.

**Table 2:** Comparison of diagnosis rate between mNGS group and non-mNGS group.

Group	Case (*n*)	Etiological diagnosis	Bacteria	Fungi	Virus	Mixed infection
mNGS	124	115 (92.7)	38 (30.6)	70 (56.5)	34 (27.4)	30 (24.2)
Non-mNGS	163	113 (69.3)	29 (17.8)	74 (45.4)	21 (12.9)	12 (7.4)
*χ* ^2^		23.646	6.502	3.441	9.606	15.971
*p*		<0.001	0.011	0.064	0.002	<0.001

mNGS: metagenomic next-generation sequencing.

**Table 3:** Positive rate of the detection methods.

Methods	Sample case (*n*)	Positive case (*n*)	Positive rate (%)
SC	281	133	47.3
CM	245	111	45.3
mNGS	124	104	83.9
PS	207	40	19.3

SC, secretion culture; CM: corneal laser confocal microscopy; PS: pathological section; mNGS, metagenomic next-generation sequencing.

**Table 4:** Treatment of 287 infectious keratitis.

Treatment methods	Case (*n*)	Percentage (%)
Corneal debridement and stromal anterior chamber drug injection	141	49.1
Secondary keratoplasty	101	35.2
Keratoplasty	102	35.5
Secondary vitrectomy	3	1.0
Secondary enucleation of ocular contents	3	1.0
Drug irrigation and conjunctival flap covering	10	3.5
Secondary enucleation of ocular contents	1	0.3
Enucleation of ocular contents	10	3.5
Lacrimal canaliculotomy and palpebral fissure	1	0.3
Unoperated	23	8.0
Total	287	100

## Data Availability

All data generated or analyzed during this study are included in the manuscript.

## Abbreviations

mNGS: Metagenomic next-generation sequencing
IK: Infectious keratitis
SC: Secretion culture
CM: Confocal microscopy
PS: Pathological section
FK: Fungal keratitis
KP: Keratoplasty
ECE: Enucleation of eye contents
BK: Bacterial keratitis
PK: Parasitic keratitis.

## Additional Points

*Highlights*. (1) Trauma and foreign matter were the main cause of IK. (2) The positivity rate of mNGS for bacteria and viruses was higher than that of the other methods. (3) mNGS is appropriate for mixed infections in IK.

## Supplementary Materials

Supplementary Materials Supplementary 1. Table S1: Pathogenic causes of 287 infectious keratitis (IK) [*n* (%)]. Supplementary 2. Table S2: Pathogenic fungal profile of 144 fungal keratitis (Secretion culture/metagenomic next-generation sequencing, [*n* (%)]). Supplementary 3. Table S3: Pathogenic bacterial profile of 67 bacterial keratitis (Secretion culture/metagenomic next-generation sequencing, [*n* (%)]). Supplementary 4. Table S4: Pathogenic viral profile of 55 viral keratitis (metagenomic next-generation sequencing, *n*), and pathogenic parasitic profile of 4 parasitic keratitis (corneal laser confocal microscopy/metagenomic next-generation sequencing, *n*).

## References

[1] Thomas P. A., Geraldine P. (2007). Infectious keratitis. *Current Opinion in Infectious Diseases*.

[2] Ung L., Bispo P. J., Shanbhag S. S., Gilmore M. S., Chodosh J. (2019). The persistent dilemma of microbial keratitis: global burden, diagnosis, and antimicrobial resistance. *Survey of Ophthalmology*.

[3] Ting D. S. J., Ho C. S., Deshmukh R., Said D. G., Dua H. S. (2021). Infectious keratitis: an update on epidemiology, causative microorganisms, risk factors, and antimicrobial resistance. *Eye*.

[4] Henry C. R., Flynn H. W., Miller D., Forster R. K., Alfonso E. C. (2012). Infectious keratitis progressing to endophthalmitis: a 15-year study of microbiology, associated factors, and clinical outcomes. *Ophthalmology*.

[5] Al-Mujaini A., Al-Kharusi N., Thakral A., Wali U. K. (2009). Bacterial keratitis: perspective on epidemiology, clinico-pathogenesis, diagnosis and treatment. *Sultan Qaboos Univ Med J*.

[6] Shah S., Wozniak R. A. F. (2023). *Staphylococcus aureusPseudomonas aeruginosa*. *Frontiers in Cellular and Infection Microbiology*.

[7] Soleimani M., Cheraqpour K., Sadeghi R., Pezeshgi S., Koganti R., Djalilian A. R. (2023). Artificial intelligence and infectious keratitis: where are we now?. *Life*.

[8] Brown L., Leck A. K., Gichangi M., Burton M. J., Denning D. W. (2021). The global incidence and diagnosis of fungal keratitis. *The Lancet Infectious Diseases*.

[9] Tian L., Cao W., Yue R. (2019). Pretreatment with Tilianin improves mitochondrial energy metabolism and oxidative stress in rats with myocardial ischemia/reperfusion injury via AMPK/SIRT1/PGC-1 alpha signaling pathway. *Journal of Pharmacological Sciences*.

[10] Gu W., Miller S., Chiu C. Y. (2019). Clinical metagenomic next-generation sequencing for pathogen detection. *Annual Review of Pathology: Mechanisms of Disease*.

[11] Huang J., Jiang E., Yang D. (2020). Metagenomic next-generation sequencing versus traditional pathogen detection in the diagnosis of peripheral pulmonary infectious lesions. *Infection and Drug Resistance*.

[12] Huang Z.-D., Zhang Z.-J., Yang B. (2020). Pathogenic detection by metagenomic next-generation sequencing in osteoarticular infections. *Frontiers in Cellular and Infection Microbiology*.

[13] Borroni D., Bonzano C., Sánchez-González J. M. (2023). Shotgun metagenomic sequencing in culture negative microbial keratitis. *European Journal of Ophthalmology*.

[14] Seitzman G. D., Thulasi P., Hinterwirth A., Chen C., Shantha J., Doan T. (2019). Capnocytophaga keratitis: clinical presentation and use of metagenomic deep sequencing for diagnosis. *Cornea*.

[15] Greenwald M. F., Redd T. K., Doan T., McLeod S. D., Seitzman G. D. (2022). Very late onset LASIK flap Acremonium fungal keratitis confirmed by metagenomic deep sequencing. *American journal of ophthalmology case reports*.

[16] Xu S., Lu S., Gu Y. (2023). Metagenomic next-generation sequencing to investigate infectious keratitis by Corynespora cassiicola: a case report. *Frontiers of Medicine*.

[17] Gu M., He P., Lyu C. (2019). Spinosin and 6’’’Feruloylspinosin protect the heart against acute myocardial ischemia and reperfusion in rats. *Molecular Medicine Reports*.

[18] Sharma S., Kunimoto D. Y., Gopinathan U., Athmanathan S., Garg P., Rao G. N. (2002). Evaluation of corneal scraping smear examination methods in the diagnosis of bacterial and fungal keratitis: a survey of eight years of laboratory experience. *Cornea*.

[19] Jalbert I., Stapleton F., Papas E., Sweeney D. F., Coroneo M. (2003). In vivo confocal microscopy of the human cornea. *British Journal of Ophthalmology*.

[20] Hudson J., Al-Khersan H., Carletti P., Miller D., Dubovy S. R., Amescua G. (2022). Role of corneal biopsy in the management of infectious keratitis. *Current Opinion in Ophthalmology*.

[21] Gu W., Miller S., Chiu C. Y. (2019). Clinical metagenomic next-generation sequencing for pathogen detection. *Annual Review of Pathology: Mechanisms of Disease*.

[22] Chiu C. Y., Miller S. A. (2019). Clinical metagenomics. *Nature Reviews Genetics*.

[23] Deng Y., Wen X., Hu X. (2020). Geographic difference shaped human ocular surface metagenome of young han Chinese from Beijing, wenzhou, and guangzhou cities. *Investigative Ophthalmology and Visual Science*.

[24] Aragona P., Baudouin C., Benitez Del Castillo J. M. (2021). The ocular microbiome and microbiota and their effects on ocular surface pathophysiology and disorders. *Survey of Ophthalmology*.

[25] Löffler J., Hebart H., Schumacher U., Reitze H., Einsele H. (1997). Comparison of different methods for extraction of DNA of fungal pathogens from cultures and blood. *Journal of Clinical Microbiology*.

[26] Dong Q., Brulc J. M., Iovieno A. (2011). Diversity of bacteria at healthy human conjunctiva. *Investigative Ophthalmology and Visual Science*.

[27] Prashanthi G. S., Jayasudha R., Chakravarthy S. K. (2019). Alterations in the ocular surface fungal microbiome in fungal keratitis patients. *Microorganisms*.

[28] Ge C., Wei C., Yang B. X., Cheng J., Huang Y. S. (2019). Conjunctival microbiome changes associated with fungal keratitis: metagenomic analysis. *International Journal of Ophthalmology*.

[29] Ung L., Bispo P. J. M., Shanbhag S. S., Gilmore M. S., Chodosh J. (2019). The persistent dilemma of microbial keratitis: global burden, diagnosis, and antimicrobial resistance. *Survey of Ophthalmology*.

[30] Vaddavalli P. K., Garg P., Sharma S., Sangwan V. S., Rao G. N., Thomas R. (2011). Role of confocal microscopy in the diagnosis of fungal and acanthamoeba keratitis. *Ophthalmology*.

[31] Chidambaram J. D., Prajna N. V., Palepu S. (2018). In vivo confocal microscopy cellular features of host and organism in bacterial, fungal, and acanthamoeba keratitis. *American Journal of Ophthalmology*.

[32] Cabrera‐Aguas M., Khoo P., Watson S. L. (2022). Infectious keratitis: a review. *Clinical and Experimental Ophthalmology*.

[33] Dahlgren M. A., Lingappan A., Wilhelmus K. R. (2007). The clinical diagnosis of microbial keratitis. *American Journal of Ophthalmology*.

[34] Burton M. J., Pithuwa J., Okello E. (2011). Microbial keratitis in east africa: why are the outcomes so poor?. *Ophthalmic Epidemiology*.

[35] Wallin Philippot K., Baron J., Sánchez Romano J. (2023). Infectious keratoconjunctivitis in semi-domesticated reindeer (*Rangifer tarandus* tarandus): a questionnaire-based study among reindeer herders in Norway and Sweden. *Acta Veterinaria Scandinavica*.

[36] Acharya Y., Acharya B., Karki P. (2017). Fungal keratitis: study of increasing trend and common determinants. *Nepal Journal of Epidemiology*.

[37] Acharya Y., Acharya B., Karki P. (2017). Fungal keratitis: study of increasing trend and common determinants. *Nepal Journal of Epidemiology*.

[38] Alreshidi S. O., Vargas J. M., Ahmad K. (2024). Differentiation of acanthamoeba keratitis from other non-acanthamoeba keratitis: risk factors and clinical features. *PLoS One*.

[39] Bharath N., Ge M. (2018). In vitro efficacy of Trichoderma harzianum against major fungal pathogens of Teak and Mahogony seedlings. https://www.researchgate.net/publication/326081237_In_vitro_efficacy_of_Trichoderma_harzianum_against_major_fungal_pathogens_of_Teak_and_Mahogony_seedlings.

[40] Gallon P., Parekh M., Ferrari S., Fasolo A., Ponzin D., Borroni D. (2019). Metagenomics in ophthalmology: hypothesis or real prospective?. *Biotechnology Reports*.

[41] Doan T., Wilson M. R., Crawford E. D. (2016). Illuminating uveitis: metagenomic deep sequencing identifies common and rare pathogens. *Genome Medicine*.

[42] Li W., Wang Z., Qu J., Zhang Y., Sun X. (2019). Acanthamoeba keratitis related to contact lens use in a tertiary hospital in China. *BMC Ophthalmology*.

[43] Johnston S. P., Sriram R., Qvarnstrom Y. (2009). Resistance of Acanthamoeba cysts to disinfection in multiple contact lens solutions. *Journal of Clinical Microbiology*.

[44] Rayamajhee B., Willcox M. D., Henriquez F. L., Petsoglou C., Carnt N. (2021). Acanthamoeba keratitis: an increasingly common infectious disease of the cornea. *The Lancet Microbe*.

[45] Rammohan R., Baidwal S., Venkatapathy N., Lorenzo-Morales J., Raghavan A. (2023). A 5-year review of coinfections in acanthamoeba keratitis from south India. *Eye and Contact Lens: Science and Clinical Practice*.

[46] Rayamajhee B., Sharma S., Willcox M. (2022). Assessment of genotypes, endosymbionts and clinical characteristics of Acanthamoeba recovered from ocular infection. *BMC Infectious Diseases*.

[47] Rayamajhee B., Willcox M., Henriquez F. L. (2024). The role of naturally acquired intracellular *Pseudomonas aeruginosa* in the development of Acanthamoeba keratitis in an animal model. *PLoS Neglected Tropical Diseases*.

