# Supplementary material for: Application of Metagenomic Next-Generation Sequencing in the Diagnosis of Infectious Keratitis
Source: J Ophthalmol. 2024 Apr 29;2024:9911979. doi: 10.1155/2024/9911979 (PMC11074721; doi:10.1155/2024/9911979)
Supplement: Supplementary Materials — Supplementary 1. Table S1: Pathogenic causes of 287 infectious keratitis (IK) [n (%)]. Supplementary 2. Table S2: Pathogenic fungal profile of 144 fungal keratitis (Secretion culture/metagenomic next-generation sequencing, [n (%)]). Supplementary 3. Table S3: Pathogenic bacterial profile of 67 bacterial keratitis (Secretion culture/metagenomic next-generation sequencing, [n (%)]). Supplementary 4. Table S4: Pathogenic viral profile of 55 viral keratitis (metagenomic next-generation sequencing, n), and pathogenic parasitic profile of 4 parasitic keratitis (corneal laser confocal microscopy/metagenomic next-generation sequencing, n). [file 9911979.f1.docx]

**Table S1.** Pathogenic causes of 287 infectious keratitis (IK) [n (%)]

| Trauma or foreign matter | **Case (%)** | Non-trauma | **Case (%)** |
| --- | --- | --- | --- |
| Plant trauma | 50 (17.32) | Insufficient eyelid closure | 7 (2.44) |
| Foreign matter/rub eyes | 28 (9.66) | Facial herpes zoster | 2 (0.70) |
| Chemical products | 22 (7.55) | Uveitis | 2 (0.70) |
| Insects | 11 (3.83) | Common cold | 2 (0.70) |
| Post operation | 11 (3.83) | Measles | 1 (0.35) |
| Lacrimal duct tube insertion | 1 (0.35) | Chronic recurrent infection | 63 (21.95) |
| Metals | 9 (3.13) | Unknown | 66 (22.99) |
| Dirty water | 4 (1.38) |  |  |
| Fireworks | 4 (1.38) |  |  |
| Trichiasis | 1 (0.35) |  |  |
| Other | 4 (1.38） |  |  |
| Total | 144 (50.17) | Total | 143 (49.83) |

**Table S2** Pathogenic fungal profile of 144 fungal keratitis (Secretion culture/ metagenomic next-generation sequencing, [n (%)]).

| **Filamentous fungi** | | **Strain (%)** | **Non-filamentous fungi** | | **Strain (%)** |
| --- | --- | --- | --- | --- | --- |
| *Fusarium*  46 (36.8%) | *Solanum* | 25 (20.0) | Yeasts  4 (3.2%) | *Candida albicans* | 3 (2.4) |
|  | *Oxysporum* | 7 (5.6) |  | *Candida parapsilosis* | 1 (0.8) |
|  | Others | 14 (11.2) | Yeast-like fungi  2 (1.6%) | *Saccharomyces cerevisiae* | 1 (0.8) |
| *Aspergillus*  31 (24.8%) | *Flavus* | 13 (10.4) |  | *Saccharomyces ovalis* | 1 (0.8) |
|  | *Fumigatus* | 7 (5.6) |  | |  |
|  | *Polypolyus* | 3 (2.4) |  | |  |
|  | Others | 8 (6.4) |  | |  |
| *Alternaria*  6 (4.8%) | *Alternata* | 4 (3.2) |  | |  |
|  | Others | 2 (1.6) |  | |  |
| *Penicillium*  5 (4.0%) | *Flavus* | 2 (1.6) |  | |  |
|  | Others | 3 (2.4) |  | |  |
| *Cladosporium*  5 (4.0%) | *Complex group* | 5 (4.0) |  | |  |
| *Paecilomyces* | | 3 (2.4) |  | |  |
| *Curvularia* | | 3 (2.4) |  | |  |
| *Ocher Rosamson complex group* | | 2 (1.6) |  | |  |
| *Pythium insidiosum* | | 2 (1.6) |  | |  |
| *Erysiphe* | | 1 (0.8) |  | |  |
| *Colletotrichum* | | 1 (0.8) |  | |  |
| *Corynespora* | | 1 (0.8) |  | |  |
| *Trichophyton* | | 1 (0.8) |  | |  |
| Unclassified | | 11 (8.8) |  | |  |
| **Total** | | **119 (94.4)** | **Total** | | **6(5.6)** |

**Table S3** Pathogenic bacterial profile of 67 bacterial keratitis (Secretion culture/ metagenomic next-generation sequencing, [n (%)]).

| **G- bacteria** | | **Strain (%)** | **G+ bacteria** | | **Strain (%)** |
| --- | --- | --- | --- | --- | --- |
| *Pseudomonas*  15 (19.7%) | *Aeruginosa* | 9 (11.8) | *Streptococcus*  14 (18.4%) | *Pneumoniae* | 8 (10.5) |
|  | Others | 6 (7.9) |  | *Sanguis* | 2 (2.6) |
| *Moraxella* | | 3 (3.9) |  | Others | 4 (5.3) |
| *Acinetobacter* | | 3 (3.9) | *Staphylococcus*  13 (17.1%) | *Epidermidis* | 8 (10.5) |
| *Prevotella* | | 3 (3.9) |  | *Aureus* | 5 (6.6) |
| *Codiophilus* | | 2 (2.6) | *Bacillus SPP* | | 3 (3.9) |
| *Xanthomonas* | | 2 (2.6) | *Nocardia* | | 3 (3.9) |
| *Escherichia* | | 2 (2.6) | *Corynebacterium* | | 2 (2.6) |
| *Klebsiella* | | 2 (2.6) |  | |  |
| *Delftia* | | 2 (2.6) |  | |  |
| *Enterobacterium* | | 1 (1.3) |  | |  |
| *Cuprophilus* | | 1 (1.3) |  | |  |
| *Aureus* | | 1 (1.3) |  | |  |
| *Cardiobacterium* | | 1 (1.3) |  | |  |
| *Serratia* | | 1 (1.3) |  | |  |
| *Morganella* | | 1 (1.3) |  | |  |
| *Acanthobacterium* | | 1 (1.3) |  | |  |
| **Total** | | **41 (53.9)** | **Total** | | **35 (46.1)** |

**Table S4** Pathogenic viral profile of 55 viral keratitis (metagenomic next-generation sequencing, n), and pathogenic parasitic profile of 4 parasitic keratitis (corneal laser confocal microscopy/ metagenomic next-generation sequencing, n).

| **Type** | | **Strain (n)** | **Percentage (%)** |
| --- | --- | --- | --- |
| **Virus strain** | HSV-1 | 31 | 96.9 |
|  | VZV | 1 | 3.1 |
|  | **Total** | **32** | **100** |
| **Parasite strain** | *Acanthamoeba* | 3 | 75 |
|  | *Nematode* | 1 | 25 |
|  | **Total** | **4** | **100** |
